# Hypertonic saline versus mannitol for brain relaxation in supratentorial tumor surgery: a prospective randomized trial

**DOI:** 10.1016/j.bjane.2025.844684

**Published:** 2025-09-16

**Authors:** Eren Fatma Akcil, Ozlem Korkmaz Dilmen, Yusuf Tunali

**Affiliations:** University of Istanbul-Cerrahpasa, Cerrahpasa Faculty of Medicine, Department of Anesthesiology and Reanimation, Bakırkoy, Istanbul

**Keywords:** Craniotomy, Glioblastoma, Mannitol

## Abstract

**Background:**

Hypertonic saline and mannitol are widely used to improve brain relaxation during supratentorial mass surgeries. Although continuous administration of hypertonic saline is known to reduce intracranial pressure, it has not yet been evaluated in supratentorial mass surgeries.

**Methods:**

After institutional ethical committee approval, 92 patients scheduled for supratentorial craniotomy with glioblastoma multiforme, metastasis and/or midline shift (> 0.5 cm) were enrolled into this prospective, randomized, and double-blind study. The patients received hypertonic saline 3 mL.kg^-1^ bolus, hypertonic saline infusion 20 mL.h^-1^ or 20 % mannitol 0.6 gr.kg^-1^ after head positioning. Brain relaxation score (1 = Perfectly relaxed, 2 = Satisfactorily relaxed, 3 = Firm brain and 4 = Bulging brain) was the primary outcome. Sodium and chlorine levels were the secondary outcomes. Postoperative brain edema and midline shift were assessed.

**Results:**

After randomization, two patients were excluded from the study. Brain relaxation scores were higher with hypertonic saline bolus compared to mannitol (*p* = 0.047). The effect size between groups for brain relaxation score was 0.22. Hypertonic saline continuous infusion and mannitol were similar with respect to brain relaxation scores. Sodium and chlorine levels were lower in the mannitol group. Postoperative midline shift and edema were lower with continuous hypertonic saline compared to other groups (*p* = 0.001, *p* = 0.006).

**Conclusion:**

Continuous infusion of 3 % hypertonic saline was associated with better relaxation scores in the intraoperative period and with lower incidences of edema/midline shift in the postoperative period of supratentorial mass surgeries with glioblastoma multiforme, metastasis and/or midline shift.

## Introduction

One of the main goals of neuroanesthesia is to ensure satisfactory relaxation of the brain during supratentorial mass surgery. Surgical retraction also contributes to vasogenic edema and increased intracranial pressure caused by the tumor.^1^

Hyperosmolar therapy is frequently used in the perioperative period to increase brain elastance and decrease brain edema and intracranial pressure. While the efficacies of 3 % Hypertonic Saline (HS) and 20 % mannitol used for this purpose were found to be similar in some studies, other studies showed that hypertonic saline was more effective.^2^, ^3^, ^4^ As a result, no consensus has yet been reached regarding the dose and duration of administration of HS and more studies are needed on this subject.^1^^,^^2^^,^^4^^,^^5^

Brain swelling has been reported more frequently after opening the dura during surgery of supratentorial masses diagnosed as glioblastoma multiforme, metastasis and/or with midline shift.^6^

Continuous administration of HS in traumatic brain injury patients with increased intracranial pressure has been studied and has been shown to increase survival.^7^ Moreover, expected electrolyte disturbances could be milder compared to bolus dosing of HS. Renal damage due to hyperchloremia and metabolic acidosis are the points to be considered in long-term use of HS. Continuous administration of HS has not been studied yet in supratentorial mass surgery. The primary aim of this prospective, randomized, double-blind study was to compare the effects of continuous infusion of HS with bolus administration of 3 % HS and 20 % mannitol on brain relaxation and the secondary aim was to compare the effects on serum electrolyte levels during supratentorial mass surgery with glioblastoma multiforme, metastasis and/or midline shift.

## Materials and methods

The study was registered on Clinical Trials.gov on March 15, 2020 (NCT 04,314,674). The procedures performed in this study were approved by the ethical committee of the University of Istanbul-Cerrahpasa (Ethical Committee n° 05712/2019-186156) and adhere to the 1964 Helsinki Declaration and its later amendments or comparable ethical standards. This prospective, randomized and double-blind study was performed from April 2020 to December 2022 on a total of 92 patients with glioblastoma multiforme, metastasis and/or intracranial midline shift (> 0.5 cm) scheduled for supratentorial mass resection, aged 18‒70 years, Glasgow Coma Scale (GCS): ≥ 13 and ASA (American Society of Anesthesiologists) I‒III class. The midline shift was measured in the axial plan of the cranial tomography at the level of the foramen of Monro, which is the channel connecting the frontal horns of the lateral ventricles to the third ventricles, by first measuring the width of the intracranial space (“a”), followed by measuring the distance from the bone to the septum pellucidum (“b”), and then the midline shift determined by (a/2) ‒ b. Written informed consent was obtained from all patients. Patients with renal failure, congestive heart failure and fluid-electrolyte imbalance (cerebral salt loss, diabetes insipidus, inappropriate antidiuretic hormone secretion) were excluded.

Patients were premedicated with 0.05 mg.kg^-1^ Intravenous (IV) midazolam and taken to the operating room. Electrocardiography, noninvasive arterial pressure, Peripheral Oxygen Saturation (SpO_2_), Bispectral Index (BIS) and electroencephalographic Density Spectral Array (DSA) were monitored in the operating room. Propofol (1‒2 mg.kg^-1^ IV), rocuronium (0.15 mg.kg^-1^ IV) and remifentanil (0.1 µg.kg^-1^min^-1^) IV infusion were used for induction of anesthesia. Sevoflurane inhalation in an oxygen-air mixture with FiO_2_ of 35 % was used for maintenance of anesthesia. Sevoflurane concentration was titrated between 0.5‒1 MAC according to BIS and DSA. Remifentanil maintenance began at a dose of 0.05‒0.1 µg.kg^-1^min^-1^ and titrated to maintain ± 20 % of the initial Mean Arterial Pressure (MAP). After orotracheal intubation patients were ventilated with volume-controlled mode, tidal volume 8 mL.kg^-^^1^ (ideal body weight), inspiration: expiration ratio of 1:2, Positive End-Expiratory Pressure (PEEP) 5 cm H_2_O and the respiratory rate (10–12 per minute) was adjusted to maintain PaCO_2_ in the range of 35 to 38 mmHg. Each patient underwent invasive arterial pressure monitoring with a radial arterial cannula, end-tidal carbon dioxide pressure monitoring, and diuresis was monitored with a urinary catheter. Each patient received isolen-s solution 2‒3 mL.kg.^-^^1^.h^-1^ IV.

Each patient underwent scalp block with 0.5 % bupivacaine with a maximum dose of 2 mg.kg^-^^1^. After the pin head holder application, the head was positioned with 30 degrees of elevation. Head rotation was maximum 45 degrees and recorded (Neutral, 0°‒30°, 30°‒45°). All patients were given 4 mg ondansetron IV as an antiemetic during bone flap placement. Patients were extubated after decurarization with sugammadex (2 mg.kg^-1^) at the end of surgery. All patients were followed up in the neurosurgical intensive care unit in the first 24 h postoperatively.

The study was performed in three groups: Group 1: 3 % NaCl (HS) 3 mL.kg^-1^ IV bolus; Group 2: 3 % NaCl (HS) Continuous infusion 20 mL.h-^1^; Group 3: 20 % mannitol 0.6 gr.kg^-1^ IV bolus.

Patient groups were determined by the closed envelope method. After head fixation, HS or mannitol infusions were started in all patients. In Groups 1 and 3, HS and mannitol infusions were administered in 20 min. In Group 2, HS infusion was continued until the end of surgery. HS and mannitol were prepared by the anesthesia nurse (the bags of the solutions were closed so that the writings were not visible). In Groups 1 and 3, arterial blood gas samples were taken before HS and mannitol were administered (Baseline) and at the 30th minute after the end of infusion and then at the 2nd and 4th hour; in Group 2, arterial blood gas samples were taken before HS was administered and at the 30th minute after the infusion started and then at the 2nd and 4th hour. Sodium, chlorine, base excess, lactate levels and osmolarity (calculated) were recorded. Arterial blood gas analyzer (ABL800 FLEX, Radiometer®, Denmark) was used for sodium, chlorine, base excess, lactate and osmolarity levels.

When the dura was opened by the surgical team, brain relaxation was evaluated by the surgeon on a 4-point scale (1 = Perfectly relaxed, 2 = Satisfactorily relaxed, 3 = Firm brain and 4 = Bulging brain) by looking at the relationship between the brain and the dura. To minimize bias in BRS assessment, 2 neurosurgeons decided BRS without the knowledge of the other surgeon's decision. The neurosurgeons who decided BRS were blinded to group allocation.

Demographic data, preoperative steroid use, mass location, pathological diagnosis, the position in which the operation was performed, the degree of head rotation and the duration of the operation were recorded. Total urine output and fluid balance were recorded at the end of the operation. The presence or absence of a midline shift (> 0.5 cm) and edema on cranial CT in the first six hours postoperatively was recorded (0 = Absent, 1 = Exist). The midline shift was quantified using the same method of the preoperative measurement described above. Postoperative edema was decided as either existing (1) or absent (0) and it was considered existing when there were areas of low density and loss of gray/white matter differentiation, on an unenhanced image. The obliteration of the cisterns and sulcal spaces were also evaluated. In the study, the patient, the surgeon evaluating brain relaxation and the anesthesiologist evaluating the postoperative CT did not know which hyperosmolar agent was used.

If serum Na level reached 155 meq.L^-1^ and Cl^-1^ reached 110 mmoL.L^-1^, hypertonic saline administration was discontinued. Preoperative and postoperative urea and creatinine values were recorded.

The rules applied for stopping the study: Unconsciousness after surgery, serum sodium levels > 155 meq.L^-1^ and chlorine levels >110 mmoL.L^-1^.

The patients were closely monitored neurologically in the postoperative period for the possibility of central pontine myelinolysis as an adverse event. Postoperative cranial MR imaging findings were evaluated.

The data analysis of the study followed a per-protocol approach.

The primary endpoint of this study was to compare the effects of continuous infusion with bolus administration of 3 % HS and 20 % mannitol on brain relaxation and the secondary endpoint was to compare the effects on serum electrolyte (sodium, chlorine) levels in the surgery of supratentorial masses with glioblastoma multiforme, metastasis and/or midline shift.

### Statistical analysis

A difference of 1-point in BRS between the groups was considered primary endpoint for the power analysis.^2^ The power analysis was performed with the G*Power statistical program (ver. 3.1.9.7). A total of 84 patients (28 subjects in each treatment group) was calculated for a Cohen’s d effect size of 0.5 (expected mean difference of 1.0, SD in both groups of 1.2 for BRS) with a probability of error type I of 0.05 and power of 0.95. Sample size was increased to at least 30 patients per treatment group to compensate for potential dropouts and possible inaccuracy of predictions used for the power analysis. The conformity of the continuous variables in the study to normal distribution was evaluated graphically and by Shapiro-Wilks test. One-Way ANOVA test was used for comparisons between groups of parameters showing normal distribution. Kruskal Wallis non-parametric variance analysis was used for comparisons between groups of parameters not showing normal distribution. Bonferroni correction was applied in pairwise comparisons.

Mean ± Standard Deviation and median (minimum‒maximum) values were used to represent descriptive statistics.

Cross tabulations were created. Number (n), percentage (%) and Chi-Square (χ^2^) test statistics were given for the comparison of categorical variables according to groups such as gender, ASA scores, Body Mass Index (BMI), corticosteroid administration, site and pathology of the mass, preoperative presence of the midline shift, metastasis and GBM, head position, BRS and also postoperative midline shift and edema. Kruskal-Wallis non-parametric variance analysis was used for comparison of age between groups which did not show normal distribution.

One-Way ANOVA test was used for intergroup comparisons of normally distributed parameters such as baseline osmolarity levels. Kruskal-Wallis non-parametric analysis of variance was used for intergroup comparisons of parameters that did not show normal distribution such as sodium, chlorine, base excess, lactate and MAP levels at all time intervals, and osmolarity levels in the 30th min, 2nd and 4th hours, and also urine output and fluid balance. Bonferroni correction was used in pairwise comparisons, and the results of the analysis were given.

In order to examine whether the parameters in the study differed at the measurement times (baseline, 30th min, 2nd hour, 4th hour), repeated ANOVA measures were used for the parameters with normal distribution such as lactate levels in Groups 2 and 3, osmolarity levels in Groups 1 and 2 and MAP measurements in Group 2. Dependent sample Friedman's test was used for the parameters without normal distribution such as sodium, chlorine, base excess levels in all groups and lactate levels in Group 1, osmolarity levels in Group 3 and MAP measurements in Groups 1 and 3. Bonferroni correction was performed for pairwise comparisons and the results of the analysis were given.

Dependent sample *t*-test was used to compare preop-postop urea and creatinine values for normally distributed parameters and Wilcoxon Signed Rank test was used for non-normally distributed parameters.

The tests were two-sided. Data transformation was not required.

IBM SPSS Statistics 21.0 (IBM Corp. Released 2012. IBM SPSS Statistics for Windows, Version 21.0. Armonk, NY: IBM Corp.) and MS-Excel 2007 programs were used. Statistical significance level was accepted as *p* < 0.05.

## Results

**Figure 1:**
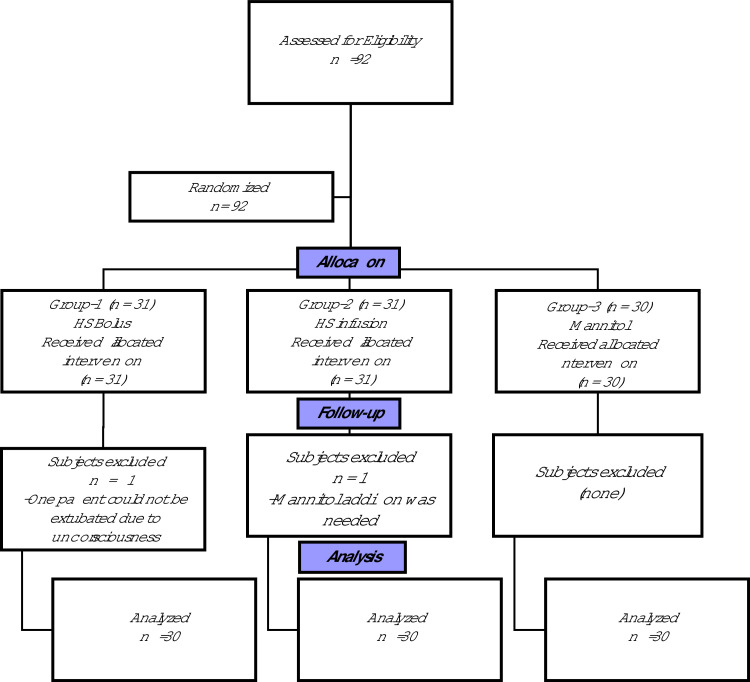
Study flowchart. HS, Hypertonic saline; n, Number.

A total of 92 patients met the inclusion criteria. In 1 patient from Group 2, mannitol addition to HS infusion was needed, and 1 patient in Group 1 could not be extubated at end of the operation due to unconsciousness, therefore they were excluded from the study. Thus, the study included 90 patients (Figure 1).

The study groups were similar with respect to gender, ASA physical status scores, Body Mass Index (BMI) and corticosteroid administration preoperatively (*p* > 0.05). Patient ages were lower in Group 3 compared to Group 2 (*p* = 0.042) (Table 1). Although there was a statistically significant difference between two groups in terms of age, we consider that given all patients were adults, it did not have a confounding effect on the study and further adjustment was not needed.

**Table 1:** Demographic data.

	Group 1 (*n* = 30)	Group 2 (*n* = 30)	Group 3 (*n* = 30)	p	MD
**Age**^b^**(years) (mean ± SD)**	51.37 ± 13.25	55.50 ± 13.59	45.40 ± 16.57	0.042^c^	Group 1 ‒ Group 2 −4.13 (−13.09‒4.82)
					Group 1 ‒ Group 3 5.97 (−2.99‒14.92)
					Group 2 ‒ Group3 10.10 (1.14‒19.06)
**Female Gender**^a^**n ( %)**	15 (50.0)	14 (46.7)	13 (43.3)	0.875	
**ASA physical status**^a^**(I/II/III) (n)**	8/19/3	5/21/4	11/17/2	0.492	
**BMI**^a^**(kg.m^-2^) (mean ± SD)**	27.76 ± 4.44	30.37 ± 5.44	27.47 ± 3.96	0.105	Group 1 ‒ Group 2 −2.61 (−5.48‒0.26)
					Group 1 ‒ Group 3 0.28 (−2.58‒3.15)
					Group 2 ‒ Group3 2.89 (0.03‒5.76)
**Preoperative corticosteroid administration**^a^**, n ( %)**	19 (63.3)	21 (70.0)	24 (80.0)	0.358	

ASA, American Society of Anesthesiologists; BMI, Body Mass Index; n, Number; SD, Standart Deviation; MD, Mean Differences.

a χ^2^.

b Kruskal-Wallis test.

c The patients ages were lower in the Group 3 comparing to Group 2 (*p* = 0.042).

The groups were similar with respect to site and pathological type of mass, surgical position, presence or not of the preoperative midline shift, metastasis and/or GBM and the duration of the operation (*p* > 0.05). The neutral head position was lower in Group 1 compared to the other groups (*p* = 0.001).

BRS3 was higher in Group 1 compared to Group 3 (*p* = 0.047) (Table 2).

**Table 2:** The brain relaxation scores, postoperative midline shift and edema.

	Group 1 (*n* = 30)	Group 2 (*n* = 30)	Group 3 (*n* = 30)	p
**BRS**^a^**I/II/III n**	1/4/25	1/10/19	0/14/16	0.047^b^
**Postoperative midline shift**^a^**, n ( %)**	20 (66.7)	7 (23.3)	18 (60.0)	0.001^c^
**Postoperative edema**^a^**, n ( %)**	21 (70.0)	10 (33.3)	19 (67.9)	0.006^d^

n, Number; BRS, Brain Relaxation Score.

a χ^2^ test.

b The BRS III were higher in the Group 1 comparing to Group 3 (*p* = 0.047).

c Postoperative midline shift was lower in the Group 2 compared to Group 1 and 3 (*p* = 0.001).

d Postoperative edema was lower in the Group 2 compared to Group 1 and 3 (*p* = 0.006).

Postoperative midline shift and edema were lower in Group 2 compared to Groups 1 and 3 (*p* = 0.001, *p* = 0.006) (Table 2).

**Figure 2:**
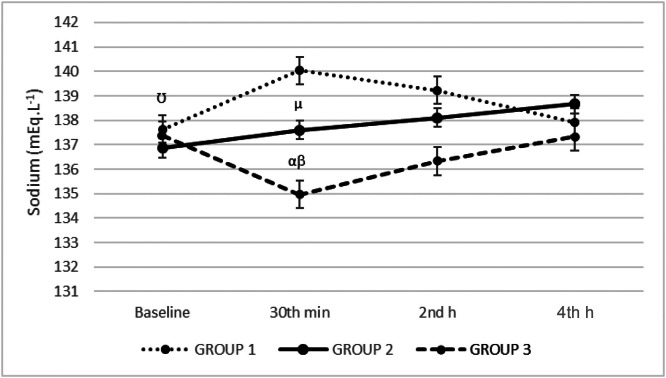
Sodium levels. (α) The sodium levels in the Group 3 were lower in the 30th min comparing to Group 1 and 2 (134.97 ± 3.46 vs. 140.03 ± 3.49 and 137.60 ± 3.64), and in the 2nd h comparing to Group 1 (136.33 ± 2.59 vs. 139.23 ± 4.01) (*p* = 0.009, *p* = 0.001 respectively). (Ʊ) Baseline sodium levels were lower comparing to 30th min, 2nd and 4th h in Group 1 (137.63 ± 3.97 vs. 140.03 ± 3.49, 139.23 ± 4.01 and 137.93 ± 3.82) (*p* < 0.001, *p* = 0.002, *p* = 0.042 respectively). (µ) The 30th min sodium levels were lower comparing to 2nd and 4th h in Group 2 (137.60 ± 3.64 vs. 138.10 ± 3.49 and 138.67 ± 3.62) (*p* = 0.001, *p* < 0.001 respectively). (β) The 30th min sodium levels were lower comparing to baseline, 2nd and 4th h in Group 3 (134.97 ± 3.46 vs. 136.33 ± 2.59 and 137.33 ± 2.52) (*p* < 0.001, *p* = 0.012 and *p* < 0.001 respectively). There was a linear increase in the sodium levels in time in the Group 2.

Sodium levels in Group 3 were lower in the 30th min compared to Groups 1 and 2 (134.97 ± 3.46 vs. 140.03 ± 3.49 and 137.60 ± 3.64), and in the 2nd hour compared to Group 1 (136.33 ± 2.59 vs. 139.23 ± 4.01) (*p* = 0.009, *p* = 0.001 respectively). Baseline sodium levels were lower compared to the 30th min, 2nd and 4th hours in Group 1 (137.63 ± 3.97 vs. 140.03 ± 3.49, 139.23 ± 4.01 and 137.93 ± 3.82) (*p* < 0.001, *p* = 0.002, *p* = 0.042 respectively). The 30th min sodium levels were lower compared to the 2nd and 4th h in Group 2 (137.60 ± 3.64 vs. 138.10 ± 3.49 and 138.67 ± 3.62) (*p* = 0.001, *p* < 0.001 respectively) and compared to baseline, the 2nd and 4th h in Group 3 (134.97 ± 3.46 vs. 136.33 ± 2.59 and 137.33 ± 2.52) (*p* < 0.001, *p* = 0.012 and *p* < 0.001 respectively). There was a linear increase in the sodium levels in time in Group 2 (Figure 2).

**Figure 3:**
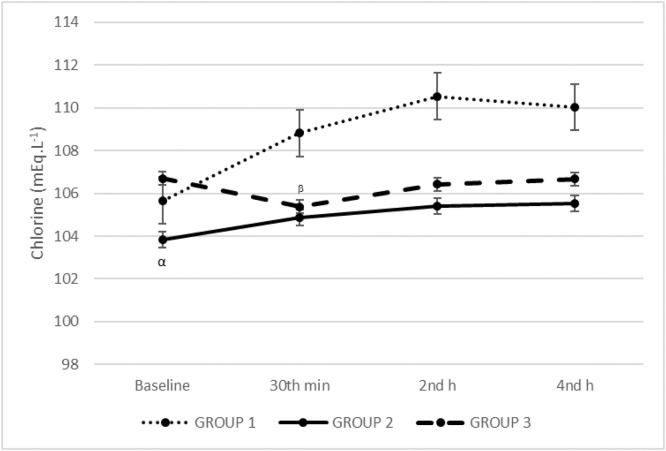
Chlorine levels. The chlorine levels were similar between groups. (α) Baseline chlorine levels were lower comparing to 30th min, 2nd and 4th h in Group 1 and Group 2 (*p* < 0.001). (β) The 30th min chlorine levels were lower comparing to baseline, 2nd and 4th h in Group 3 (*p* < 0.001). There was a linear increase in the chlorine levels in time in the Group 2.

The chlorine levels were similar between groups. Baseline chlorine levels were lower compared to the 30th min, 2nd and 4th h in Group 1 and Group 2 (*p* < 0.001). There was a linear increase in the chlorine levels in time in Group 2. The 30th min chlorine levels were lower compared to baseline, 2nd and 4th h in Group 3 (*p* < 0.001) (Figure 3).

The base excess levels were more negative in the 30th min in Group 1 compared to Group 2 (*p* = 0.032). Moreover, the base excess levels were more negative in the 30th min, 2nd and 4th h compared to baseline levels in all groups and tended to be less negative in time.

The lactate levels were similar between groups. The lactate levels were higher in the 30th min and 2nd h compared to baseline in Group 3 (*p* = 0.009, *p* = 0.038 respectively).

The group-by-time interaction effects of the parameters were for sodium *p* < 0.001, chlorine *p* < 0.001, lactate *p* = 0.002, base excess *p* = 0.086.

The osmolarity levels were lower in the 30th min in Group 3 compared to Groups 1 and 2 (*p* = 0.001, *p* = 0.004 respectively). Baseline osmolarity levels were lower compared to the 30th min, 2nd and 4th h in Group 1, and 2nd and 4th h in Group 2 (*p* < 0.05). The 30th min osmolarity levels were lower compared to baseline, 2nd and 4th h in Group 3 (*p* < 0.001, *p* = 0.042, *p* < 0.001 respectively). The groups were similar with respect to MAP’s.

The groups were similar with respect to urine output, fluid balance, preoperative and postoperative urea and creatinine levels. Postoperative creatinine levels were lower compared to preoperative ones in Groups 1 and 3 (*p* = 0.004 and *p* = 0.013 respectively) (Table 3).

**Table 3:** Urine output, fluid balance, pre- and postoperative urea and creatinine levels.

	Group 1 (*n* = 30)	Group 2 (*n* = 30)	Group 3 (*n* = 30)	p
**Urine output**^b^**(mL) (mean ± SD)**	925.33 ± 673.80	770.00 ± 756.88	766.67 ± 456.06	0.312
**Fluid balance**^b^**(mL) (mean ± SD)**	988.33 ± 760.18	1028.00 ± 796.89	827.00 ± 443.06	0.713
**Preoperative urea**^b^**(mg.dL^-1^) (mean ± SD)**	38.23 ± 14.16	41.13 ± 16.89	37.17 ± 14.46	0.787
**Postoperative urea**^b^**(mg.dL^-1^) (mean ± SD)**	44.63 ± 17.43^c^	44.77 ± 14.09^c^	37.40 ± 15.29^d^	0.281
	*c* = 1.430;	*c* = 0.957;	*d* = 0.139;	
	*p* = 0.153	*p* = 0.339	*p* = 0.891	
**Preoperative creatinine**^b^**(mg.dL^-1^) (mean ± SD)**	0.78 ± 0.14	0.80 ± 0.21	0.79 ± 0.21	0.867
**Postoperative creatinine**^b^**(mg.dL^-1^)**	0.72 ± 0.20^c^^,^^e^	0.76 ± 0.20^d^	0.73 ± 0.19^c^^,^^e^	0.366
	*c* = 2.870;	*d* = 1.608;	*c* = 2.477;	
	*p* = **0.004**	*p* = 0.119	*p* = **0.013**	

SD, Standart Deviation.

b Kruskal-Wallis test.

c Wilcoxon Signed Rank test.

d Dependent sample *t*-test.

e Postoperative cretinine levels were lower compared to preoperative ones in the Group 1 and 3 (*p* = 0.004 and *p* = 0.013 respectively).

## Discussion

This study showed that 3 % HS infusion was effective as 20 % mannitol in providing brain relaxation and better provided brain relaxation as good as 20 % mannitol and better than 3 % HS bolus administration without electrolyte disturbance, hypovolemia, hemodynamic and renal dysfunction in supratentorial masses with GBM, metastases and/or midline shift. Moreover, postoperative midline shift and edema were less in the 3 % HS infusion group.

The effect of different concentrations of mannitol and HS on brain relaxation in supratentorial craniotomies has been compared in several studies and no consensus has been reached on which is better in terms of brain relaxation.^2^, ^3^, ^4^, ^5^^,^^8^, ^9^, ^10^, ^11^, ^12^ Better brain relaxation is essential during neurosurgery and neuroanesthesia practice to improve the quality of surgical exposure, to reduce brain retractor pressure and to reduce risk of ischemia due to raised ICP. Wu et al.^4^ reported better brain relaxation with HS compared to mannitol, while no difference was found in the studies conducted by Rozet et al.,^5^ Hernandez-Palazon et al.^3^ and Fang et al.^13^ Abdulhamid et al.^14^^14^ reported statistically significant brain relaxation with HS in their meta-analysis and Mavrocordatos et al.^15^ reported better brain relaxation with HS, although not statistically significant.

In our study, we found that brain relaxation was better in the group given mannitol than in the group given echimolar HS bolus. The number of patients with neutral head position was lower in the group given HS bolus than in the other groups, but the literature has reported that the increasing effect of head rotation on intracranial pressure decreases with head elevation of 30 degrees. In addition, an increase in intracranial pressure has been reported with head rotation of more than 60 degrees or full rotation.^16^, ^17^ All patients in this study were given a head position with 30 degrees of head elevation and a maximum rotation of 45 degrees.

When we administered HS continuously, we found that it provided brain relaxation at a level close to mannitol. Studies have been performed on continuous administration of HS in traumatic brain injury cases with increased intracranial pressure, and it has been shown to decrease intracranial pressure and increase survival.^7^ With the hypothesis that the sudden increase in sodium and metabolic acidosis that may occur with HS bolus administration would be less with continuous administration, we applied HS continuously in supratentorial mass surgeries with high ICP. In sodium levels, we obtained the highest value at 30 minutes after HS bolus administration; although it decreased over time, it remained higher than baseline, while a gradual increase occurred over time in the infusion group. In the mannitol group, as expected, it was lower than in the other groups and other measurement times of the same group at 30 minutes after administration. Chlorine levels were similar to sodium. The changes in sodium and chlorine levels were consistent with other studies and within physiological limits.^11^, ^14^

In terms of metabolic acidosis, we observed that base excess levels were more negative at 30 min in the HS bolus group. In our study, unlike other studies, lactate increase in mannitol groups was not detected.^5^, ^11^ We think that this is due to the absence of increased urine output, hypovolemia and impaired fluid balance in our study, which were observed in mannitol groups in other studies.^15^

We found that HS caused an increase in osmolarity and mannitol caused a decrease in the early period. De Vivo et al.^12^ and Rozet et al.^5^ found a similar increase in osmolarity with HS and mannitol, whereas Briscoe et al.^18^ found lower osmolarity in the mannitol group at the second hour. We can explain the decrease in osmolarity with mannitol by the fact that sodium was also low during the study periods.

One of the most important side effects of HS is renal dysfunction.^19^^,^^20^ Acute kidney injury has been observed in patients with sodium levels above 155 mEq.L^-1^ and chlorine levels above 115 mEq.L^-1^ in intensive care unit patients receiving HS.^20^, ^21^ Conversely, it has been found that continuous infusion of hypertonic saline was not associated with renal dysfunction in traumatic brain injury patients due to frequent increase in renal clearance in trauma patients, which could increase the tolerance of hypertonic saline.^22^ In our study, there was no increase in the postoperative urea and creatinine levels of patients, we think due to the fact that sodium and chlorine levels were not above the specified limit.

Postoperative peritumoral edema and midline shifting are outcomes affecting neurological outcome in supratentorial tumor resection surgery.^12^ In our study, we found that HS continuous administration reduced postoperative edema and midline shift compared to mannitol and HS bolus in patients with GBM, metastases and midline shift, which constitute the risk group in this sense. Based on the results of this study, we continue to administer HS continuously for at least 24 h in the postoperative period in patients in this high-risk group.

During supratentorial craniotomies, satisfactory brain relaxation is a major challenge. Cerebral swelling has many detrimental effects resulting in poor surgical exposure, increased brain retractor pressure as a main cause of cerebral ischemia and poor neurological outcome with deficits postoperatively. In our study we achieved satisfactory brain relaxation with HS and mannitol, therefore we did not observe newly developing or worsening neurological deficits in our patients in the postoperative period.

This study has some limitations. We can suggest that ICP increases in patients with midline shift, but we could have had more objective results if ICP monitoring had been performed instead of BRS in this study. On the other hand, ICP is equivalent to the atmospheric pressure when the dura is opened. Therefore, we could not use ICP as an objective parameter to evaluate brain relaxation except before dura opening. Although BRS is a subjective scale for evaluation of brain relaxation, it is widely used in the studies investigating the effects of hyperosmolar therapy on brain bulging during craniotomies in neurosurgery and neurosurgical anesthesia fields and it is considered a significant valuation criterion for therapeutic decisions. Moreover, this scale is not validated and there could be inter-observer variability. To minimize bias in BRS assessment, two surgeons decided BRS without the knowledge of the other surgeon’s decision. Another point is that when we evaluated the amount of urine, we did not take hourly measurements over the total amount of urine, and especially the 30th minute urine amount results may have led to different results in the mannitol group. A relatively short follow-up period is another limitation of this study, the electrolyte level measurements could have been extended until the postoperative 24th hour.

## Conclusion

In supratentorial craniotomies with GBM, metastases and/or midline shift, continuous infusion of 3 % HS provided satisfactory brain relaxation and also postoperative edema and midline shift were less common compared with mannitol and HS bolus administration. Moreover, electrolyte imbalance did not develop, and renal functions were preserved. More studies addressing the effects of continuous HS on postoperative neurological outcomes and to change clinical practice of hyperosmolar therapy are needed.

## Declaration of competing interest

The authors declare no conflicts of interest.
